# Reprogramming the tumor microenvironment: synergistic mechanisms of antibody–drug conjugates and immune checkpoint inhibitors

**DOI:** 10.1093/abt/tbaf017

**Published:** 2025-09-17

**Authors:** Ling Yin, Shoubing Zhou, Hongliang Zhang, Chengbing Yao, Zaid Talal Abdulqader Al-Qadhi, Yuhua Shang, Songquan Wu, Tengchuan Jin

**Affiliations:** Center of Disease Immunity and Intervention, College of Medicine, Lishui University, Lishui 323000, China; Institute of Health and Medicine, Hefei Comprehensive National Science Center, Hefei, Anhui 230601, China; Laboratory of Structural Immunology, State Key Laboratory of Immune Response and Immunotherapy, Division of Life Sciences and Medicine, University of Science and Technology of China, Hefei 230027 China; Center of Disease Immunity and Intervention, College of Medicine, Lishui University, Lishui 323000, China; Anhui Genebiol Biotech, Ltd, Hefei 230000, China; Faculty of Medicine, University of Saba Region, Marib 14400, Yemen; Anhui Genebiol Biotech, Ltd, Hefei 230000, China; Center of Disease Immunity and Intervention, College of Medicine, Lishui University, Lishui 323000, China; Center of Disease Immunity and Intervention, College of Medicine, Lishui University, Lishui 323000, China; Institute of Health and Medicine, Hefei Comprehensive National Science Center, Hefei, Anhui 230601, China; Laboratory of Structural Immunology, State Key Laboratory of Immune Response and Immunotherapy, Division of Life Sciences and Medicine, University of Science and Technology of China, Hefei 230027 China; Anhui Genebiol Biotech, Ltd, Hefei 230000, China; Biomedical Sciences and Health Laboratory of Anhui Province, University of Science and Technology of China, Hefei 230027, China; Clinical Research Hospital of Chinese Academy of Sciences (Hefei), University of Science and Technology of China, Hefei 230001, China; Key Laboratory of Anhui Province for Emerging and Reemerging Infectious Diseases, Hefei 230027, China

**Keywords:** antibody–drug conjugates (ADCs), immune checkpoint inhibitors (ICIs), tumor microenvironment (TME), immune-related adverse events (irAEs), immune microenvironment reprogramming (IMER)

## Abstract

The integration of antibody–drug conjugates (ADCs) with immune checkpoint inhibitors (ICIs) represents a paradigm shift in oncology, combining targeted cytotoxicity and adaptive immune activation to overcome resistance in refractory tumors. This review explores their mechanistic synergy, focusing on dual functions in reprogramming the tumor immune microenvironment. ADCs mediate antibody-dependent cellular cytotoxicity (ADCC), engaging NK cells and macrophages to release tumor-associated antigens (TAAs) and damage-associated molecular patterns. Immunogenic cell death (ICD) amplifies adaptive immunity by releasing TAAs for T-cell priming, while PD-L1 upregulation creates a targetable niche for PD-1/PD-L1 inhibitors. This strategy sustains interferon-γ signaling and drives effector T-cell differentiation, but overlapping immunostimulatory signals raise risks of cytokine release syndrome and immune-related adverse events, requiring biomarker-guided risk stratification. We propose a multidimensional immune microenvironment reprogramming framework, integrating tumor-infiltrating lymphocyte phenotyping, serum biomarkers, and spatial transcriptomic mapping, to optimize ADC–ICI therapy and balance efficacy with immunopathology.

## Teaser

**Statement of Significance** This study elucidates the synergistic mechanisms of antibody–drug conjugates and immune checkpoint inhibitors in tumor microenvironment reprogramming, proposing a multidimensional immune microenvironment reprogramming framework to optimize combinatorial therapy and expand the therapeutic index for refractory cancers.

## Introduction

Cancer immunotherapy has undergone a paradigm shift with the emergence of antibody–drug conjugates (ADCs) and immune checkpoint inhibitors (ICIs) as transformative therapeutic modalities [1, 2]. These two classes of agents operate through distinct yet complementary mechanisms that offer new opportunities for combination therapy. ICIs, particularly those targeting the PD-1/PD-L1 axis, function by reinvigorating exhausted T cells and restoring antitumor immunity [3–6]. However, their clinical efficacy is frequently constrained by primary and adaptive resistance mechanisms within the tumor microenvironment (TME), including immunosuppressive cell populations and inadequate T-cell priming. Concurrently, ADCs have demonstrated remarkable precision in delivering cytotoxic payloads to tumor cells through antigen-specific targeting [7, 8]. These engineered molecules combine three critical components: (i) tumor-targeting monoclonal antibodies, (ii) stable chemical linkers, and (iii) potent cytotoxic agents. While ADCs are effective as monotherapies, they often face limitations due to heterogeneous antigen expression and complex tumor resistance networks.

The strategic integration of ADCs and ICIs represents a novel therapeutic approach that combines targeted cytotoxicity with immune checkpoint blockade. ADCs mediate their effects through two principal mechanisms: first, antibody-dependent cellular cytotoxicity (ADCC), mediated through Fcγ receptor engagement, recruits natural killer (NK) cells and macrophages to release tumor-associated antigens (TAAs) and damage-associated molecular patterns (DAMPs) [7, 8] and, second, the integration of ADCs and ICIs leverages ADC-induced antigen release and PD-L1 induction to overcome ICI resistance, creating a feedforward loop of immune activation [9, 10]. This dual mechanism creates an immunologically favorable microenvironment that complements ICI activity. Clinical validation of this approach is emerging, with combinations such as trastuzumab deruxtecan (T-DXd) plus pembrolizumab demonstrating improved responses in refractory HER2-low breast cancer and mismatch repair-deficient tumors [11, 12]. However, several challenges remain, including the management of immune-related adverse events (irAEs) and optimization of treatment sequencing [13–17]. The current biomarkers lack the precision needed to reliably predict therapeutic efficacy or toxicity, while the spatiotemporal dynamics of ADC–ICI synergy—particularly the temporal relationship between antigen release and T-cell priming—remain incompletely understood [18, 19].

This review systematically examines the mechanistic synergy between ADCs and ICIs, with particular emphasis on their complementary roles in reprogramming the TME. We introduce a novel multidimensional immune microenvironment reprogramming (IMER) framework that incorporates (i) longitudinal profiling of tumor-infiltrating lymphocytes (TILs), (ii) dynamic serum biomarker analysis [including high mobility group box 1 (HMGB1) and chemokine gradients], and (iii) spatial transcriptomic mapping of checkpoint ligand–receptor interactions. By elucidating the temporal sequence of TME modifications during ADC–ICI therapy, our model provides a precision oncology foundation for optimizing combination regimens, mitigating treatment-related toxicity, and expanding the therapeutic window for refractory malignancies. The IMER framework represents a significant advance in our understanding of ADC–ICI interactions and offers a roadmap for translating insights into improved clinical outcomes.

## Innate immune activation by ADCs as a foundational driver for tumor microenvironment reprogramming

**Figure 1:**
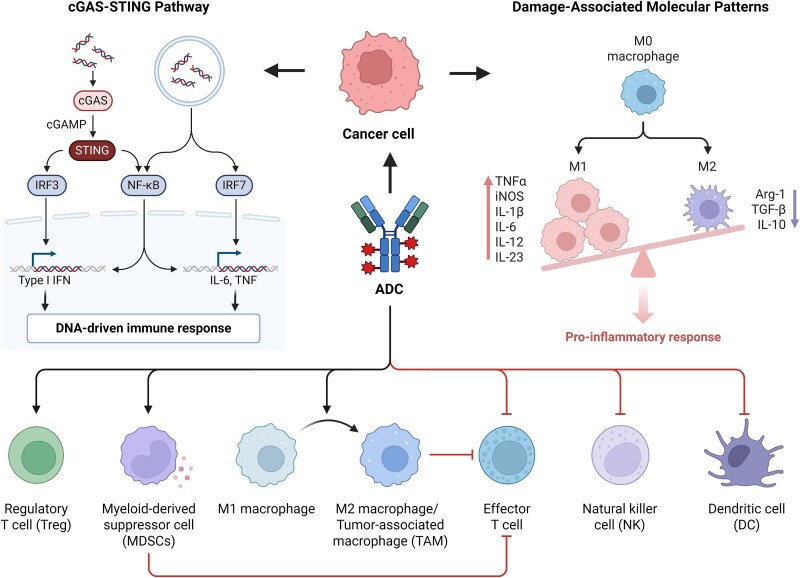
Innate immune activation by ADCs drives tumor microenvironment reprogramming. The key immune cell populations include NK cells (mediating ADCC), DCs (activated via DAMP-mediated TLR4 and mtDNA-triggered cGAS/STING pathways), effector T cells, M1/M2 macrophages, Tregs, and MDSCs.

ADC-induced tumor cell death initiates coordinated innate immune responses through DAMP and tumor antigen release, bridging innate and adaptive immunity via three core mechanisms: (i) dendritic cell/macrophage activation through pattern recognition receptors (Toll-like receptor 4 (TLR4), cyclic GMP-AMP synthase/stimulator of interferon genes (cGAS–STING)), (ii) recruitment of cytotoxic effectors (NK cells, neutrophils) with concurrent suppression of immunosuppressive populations [Tregs, myeloid-derived suppressor cells (MDSCs)], and (iii) generation of spatial heterogeneity in immune activation that critically determines therapeutic outcomes. This dynamic interplay underpins TME reprogramming and informs rational immunotherapy combinations (Fig. 1).

Following initial immune activation, secondary neutrophil extracellular trap (NET) formation occurs when IL-8 recruits neutrophils that deploy extracellular traps to capture antigens for dendritic cell uptake [20, 21]. While facilitating antigen presentation, excessive NETosis may paradoxically promote metastasis [22, 23]. Concurrently, ADC engagement of Fcγ receptors triggers ADCC-mediated lysis, releasing TAAs (HER2 ectodomains, mismatch repair proteins) and DAMPs [HMGB1, adenosine triphosphate (ATP), calreticulin (CRT)] [24–27]. These molecules activate critical pathways: HMGB1–TLR4 signaling enhances antigen presentation via NF-κB/mitogen-activated protein kinase (MAPK) activation [28–30]; ATP stimulates NOD-, LRR-, and pyrin domain-containing protein 3 (NLRP3) inflammasome-mediated IL-1β secretion to amplify Th1 responses [31–33]; and CRT promotes dendritic cell (DC) phagocytosis while enhancing CD8+ T-cell priming [34, 35].

These mechanisms collectively dismantle immunosuppressive barriers: NK cell-mediated FOXP3 cleavage depletes Tregs [36–38], while tumor necrosis factor alpha (TNF-α) suppresses MDSCs through tumor necrosis factor receptor 2 (TNFR2) signaling [36–38]. DAMP-activated DCs upregulate PD-L1 via interferon (IFN) regulatory factor 1 (IRF1), creating a feedforward loop that enhances ICI efficacy [39, 40]. Spatial distribution critically influences immune activation, with perivascular ADC accumulation driving early DAMP release and NK recruitment [41–43], while hypoxic cores remain “immunological deserts” due to poor penetration [44–49].

Beyond classical signaling, ADC-mediated lysis releases mitochondrial/cytosolic DNA that activates cGAS–STING in antigen-presenting cells (APCs) [50, 51], inducing type I IFNs that promote DC maturation and CD8+ T-cell cross-priming [52, 53]. STING activation also upregulates C-X-C motif chemokine ligand 10 (CXCL10), recruiting CD103+ DCs essential for sustained T-cell responses [54, 55]. Fc gamma receptor (FcγR) engagement polarizes macrophages toward M1 phenotypes [inducible nitric oxide synthase (iNOS)/reactive oxygen species (ROS) production] that enhance tumor killing and T-cell infiltration [56, 57], while IL-1β/TNF-α suppress IL-4/IL-13-driven M2 polarization [58, 59]. This comprehensive immune reprogramming provides the mechanistic foundation for ADC–ICI combinations.

## Adaptive immune amplification by ADC–ICI synergy: bridging innate activation to T-cell clonal expansion

**Figure 2:**
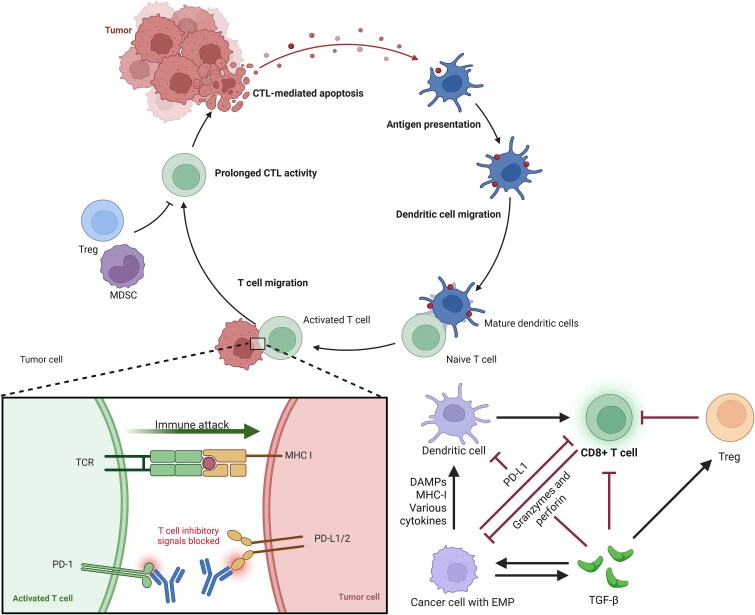
Molecular mechanisms of ADC and immune checkpoint inhibitor synergy through innate-to-adaptive immune response transition. ADCs prime the tumor microenvironment through innate immune activation, enabling ICIs to potentiate adaptive T-cell responses. The cascade of immunogenic cell death, antigen presentation, and T-cell priming establishes the mechanistic foundation for their clinical synergy.

Innate immune mechanisms triggered by ADCs—including DAMP release, DC maturation, and NK cell-mediated ADCC—prime robust adaptive immune responses when combined with ICIs. This transition relies on three critical processes: (i) cross-priming of tumor-specific T cells, (ii) PD-L1 upregulation as an adaptive resistance mechanism, and (iii) T cell receptor (TCR) clonal expansion driven by ICD-induced antigen spreading (Fig. 2).

Cross-priming of tumor-specific T cells is central to ADC–ICI synergy. DCs internalize TAAs and DAMPs (e.g. HMGB1 and ATP) released during ADC-induced ICD, undergoing maturation via TLR4/NF-κB and cGAS–STING pathways to upregulate MHC class I/II and costimulatory molecules (CD80/CD86) [60–63]. Batf3-dependent CD103+ DCs excel at cross-presenting TAAs to CD8+ T cells in tumor-draining lymph nodes, a process amplified by type I interferon (IFN-α/β) from STING activation [64–66]. This generates tumor-reactive cytotoxic T lymphocytes (CTLs) that infiltrate the TME, where their activity is potentiated by PD-1/PD-L1 blockade [67, 68]. Clinical data from HER2-targeted ADC trials link robust T-cell priming to improved ICI responses, underscoring mechanistic synergy [69–71].

However, ADC-mediated ICD releases DAMPs (e.g. HMGB1 and ATP) that activate DCs via TLR4/NF-κB, while mitochondrial DNA engages cGAS–STING to induce IFN-β. IFN-γ from activated T cells subsequently upregulates PD-L1 via Janus kinase/signal transducer and activator of transcription (JAK-STAT), creating an adaptive resistance niche exploitable by ICIs [72–76]. IFN-γ from activated CTLs and NK cells engages JAK-STAT signaling to transcriptionally induce PD-L1, limiting T-cell cytotoxicity [75, 76]. Spatial transcriptomics show heterogeneous PD-L1 expression, with prominent upregulation at invasive margins where T-cell infiltration is highest [77–81]. While this adaptation initially suppresses immunity, it establishes a therapeutic window for PD-1/PD-L1 inhibitors [82–84]. Preclinical studies demonstrate that sequential ADC–ICI treatment exploits PD-L1 upregulation as a predictive biomarker for response [85, 86], though excessive PD-L1 induction in MDSCs may exacerbate T-cell exhaustion, necessitating temporal optimization [87–89].

ADC-induced ICD also promotes TCR clonal expansion via antigen spreading, diversifying immune responses beyond initial epitopes [19, 90, 91]. Dying tumor cells release cryptic and neoantigens, which DCs cross-present to recruit polyclonal T-cell populations [92–95]. Single-cell TCR sequencing in ADC–ICI-treated patients reveals dynamic expansion of high-affinity CD8+ T cells, particularly those targeting shared tumor–testis antigens [96, 97]. ICD-associated DAMPs like CRT further enhance antigen immunogenicity by binding low-density lipoprotein receptor-related protein 1 (LRP1) on DCs, amplifying TCR signaling [98, 99]. This sustains effector T cells and generates memory precursors (TCF1+ CD8+ T cells) for durable immunity [100, 101]. Yet, excessive clonal expansion may trigger irAEs, requiring biomarker-guided strategies to balance efficacy and toxicity [102–104].

## Spatiotemporal dynamics and biomarkers for ADC–ICI synergy

The synergy between ADCs and ICIs depends on resolving the spatiotemporal dynamics of innate immune activation and adaptive immune amplification within the TME. As summarized in Table 1, serum markers (e.g. HMGB1) reflect acute immune activation, while spatial patterns (e.g. PD-L1 interface expression) predict adaptive response. Spatial heterogeneity in ADC distribution and immune cell engagement dictates temporal response patterns, with perivascular regions serving as “immune activation hubs” and hypoxic cores remaining “immunological deserts” [44]. For instance, T-DXd preferentially accumulates in HER2-low breast cancer perivascular zones, where HMGB1 release drives DC maturation via TLR4/NF-κB signaling, whereas hypoxic cores exhibit hypoxia-inducible factor 1 alpha (HIF-1α)-mediated M2 macrophage polarization and Treg expansion, creating immunosuppressive barriers [12, 105]. Single-cell RNA sequencing of sacituzumab govitecan (SG)-treated triple-negative breast cancer (TNBC) further reveals spatially distinct CD8+ T-cell clusters: DAMP-rich proximal regions harbor TCF1+ memory precursors, while distal zones are dominated by programmed cell death protein 1 high and T-cell immunoglobulin and mucin-domain containing-3 positive (PD-1hiTIM-3+) exhausted subpopulations, correlating with resistance to anti-PD-1 therapy and highlighting the need for spatial biomarkers to guide ADC–ICI sequencing [106, 107].

**Table 1:** Classification and functions of key biomarkers in ADC–ICI therapy

Category	Biomarker	Biological function	Clinical relevance
Serum biomarkers	HMGB1	Activates TLR4/NF-κB and cGAS–STING pathways; enhances antigen presentation	Predicts tumor lysis and ADC–ICI efficacy
	CXCL9/CXCL10	Recruits Batf3-dependent CD103+ DCs; promotes T-cell priming	Serum levels correlate with T-cell infiltration and ICI response
	Soluble suppression of tumorigenicity 2 (sST2)	Regulates IL-33/ST2 axis; modulates immune toxicity	Biomarker for irAE risk stratification
Spatial biomarkers	PD-L1	Interface-patterned expression enhances T-cell engagement	Predictive for ICI efficacy; stromal diffusion linked to adverse events
Liquid biopsy	ctDNA	Reflects tumor mutation burden and antigen release efficiency	Early pharmacodynamic marker for ADC activity

Multiplexed tissue imaging provides critical insights into the therapeutic response architecture. High-parameter immunofluorescence studies show that productive immune synapses—defined by CD8+ T cells engaging both PD-L1+ tumor cells and antigen-presenting DCs—are strongly linked to ADC–ICI efficacy [108, 109]. Quantitative spatial analyses further demonstrate that PD-L1 expression patterns predict outcomes: interface-patterned induction correlates with response, while stromal diffusion associates with adverse events, underscoring the need for standardized, multiregion biopsies in clinical trials [110–112].

Temporal biomarkers elucidate dynamic shifts in ADC–ICI cooperativity. Serum HMGB1 levels reflect tumor cell lysis and ICD-driven DC activation, whereas CXCL9/CXCL10 gradients predict Batf3-dependent CD103+ DC migration and T-cell priming [63, 113, 114]. Clinically, sustained chemokine elevation beyond the acute phase correlates with improved response rates, though optimal temporal cutoffs require further validation [115, 116]. Parallel ctDNA monitoring shows that reductions in tumor-associated mutations correlate with antigen release efficiency, offering a pharmacodynamic measure of ADC activity [117–119].

Integrating multidimensional assays—ctDNA kinetics, serum proteomics, and digital pathology—could enhance response prediction beyond radiographic assessment alone, though prospective validation is needed to define accuracy metrics. Machine learning approaches show promise for personalized therapy guidance, pending standardization of analytical pipelines. Future efforts must address assay harmonization and expand validation across tumor types to fully realize the potential of spatiotemporal biomarker-guided ADC–ICI combinations.

## Clinical translation and challenges of ADC–ICI combinations

The preclinical rationale for ADC–ICI synergy has driven rapid clinical development, with trials demonstrating enhanced responses in refractory malignancies. However, three major challenges hinder clinical translation: (i) optimizing dosing/sequencing to balance efficacy and toxicity; (ii) identifying predictive biomarkers for patient selection; and (iii) managing unique irAEs.

DESTINY-PanTumor02 (NCT04482309) is a phase II trial evaluating T-DXd in HER2-expressing solid tumors across seven tumor-specific cohorts (part 1) and five biomarker-driven cohorts (part 2), aiming to expand its therapeutic scope beyond current approvals [120, 121]. Meanwhile, KEYNOTE-355 (NCT02819518), a phase III study, established pembrolizumab (pembro) plus chemotherapy as standard for PD-L1-high [Combined Positive Score (CPS) ≥ 10] metastatic TNBC, though benefits were limited to this subgroup [hazard ratio (HR) = 0.65, 95% CI 0.49–0.86] [122, 123]. These trials highlight divergent strategies—DESTINY-PanTumor02’s biomarker-flexible, single-arm design versus KEYNOTE-355’s rigorous PD-L1 stratification—reflecting the tension between exploratory adaptability and confirmatory standardization in ADC–ICI development (Table 2).

**Table 2:** Head-to-head comparison of DESTINY-PanTumor02 and KEYNOTE-355: key design features, outcomes, and clinical implications in advanced solid Tumors

Dimension	DESTINY-PanTumor02	KEYNOTE-355
Trial registration	NCT04482309 (ClinicalTrials.gov)	NCT02819518 (ClinicalTrials.gov)
Study Design	Open-label, multicenter, multicohort, phase II	Randomized, double-blind, phase III
Primary objective	Explore efficacy/safety of T-DXd in HER2-expressing solid tumors (nonbreast/gastric)	Confirm efficacy/safety of pembrolizumab + chemotherapy vs chemotherapy in TNBC
Phase	Phase II (exploratory)	Phase III (confirmatory)
Target population	HER2-expressing tumors (IHC 3+/2+/1+): bladder, biliary tract, cervical, etc.	Locally recurrent inoperable/metastatic TNBC (chemotherapy-naïve)
Intervention	Trastuzumab deruxtecan (T-DXd, HER2 ADC)	Pembrolizumab (PD-1 inhibitor) + chemotherapy (nab-paclitaxel/paclitaxel/gemcitabine + carboplatin)
Control group	No control (single-arm design)	Placebo + chemotherapy
Key biomarkers	HER2 IHC 3+/2+ (nonbreast/gastric/colorectal) or HER2 IHC 2+/1+ (endometrial/ovarian/cervical)	PD-L1 CPS ≥ 1 or ≥10
Endpoints	Primary: ORR (BICR) Secondary: PFS, DOR, OS, safety	Primary: PFS (BICR), OS (all/PD-L1 subgroups) Secondary: DOR, ORR, safety
Treatment mechanism	Antibody–drug conjugate (HER2-targeted cytotoxic payload)	Immune checkpoint inhibition (PD-1) + chemotherapy
Key inclusion criteria	HER2-expressing solid tumors (excl. Breast/gastric/colorectal), measurable disease	TNBC, no prior systemic therapy
Geographic Scope	Global (multicohort, diverse tumor types)	Global (multiple centers)
Limitations	Single-arm design; requires phase III validation	Limited to TNBC; PD-L1 biomarker may restrict eligibility
Clinical impact	Demonstrated T-DXd activity in HER2-low/expression-diverse tumors	Established pembrolizumab + chemo as standard for PD-L1+ TNBC

Abbreviations: IHC, immunohistochemistry (used to assess HER2 expression levels); ORR, overall response rate (proportion of patients with tumor shrinkage); DOR, duration of response (time from response onset to disease progression/relapse); OS, overall survival (time from treatment initiation to death from any cause).

Despite promising results, not all patients benefit equally, necessitating improved biomarkers. While HER2/erb-b2 receptor tyrosine kinase 2 (ERBB2) amplification and the PD-L1 CPS show predictive value, their applicability is limited [124, 125]. Emerging markers include elevated CXCL9/CXCL10 (indicative of T-cell priming) [115, 126] and interface-patterned PD-L1 expression (superior to diffuse stromal staining) [127–129]. Early ctDNA clearance post-ADC therapy may also predict ICI response [130–132]. A composite approach integrating TIL density, chemokine gradients, and spatial immune mapping is likely optimal.

The immunostimulatory effects of ADC–ICI combinations increase toxicity risks. Cytokine release syndrome (CRS) (driven by interleukin-6 (IL-6)/IFN-γ) has occurred with brentuximab vedotin plus nivolumab, sometimes requiring IL-6 blockade [133, 134]. Pneumonitis risk (particularly with T-DXd + PD-1 inhibitors) is nearly doubled versus monotherapy, mandating early corticosteroids [135, 136]. Overlapping hematologic toxicities (e.g. ADC-induced neutropenia + ICI-induced cytopenias) complicate treatment [137, 138]. Sequential dosing (delaying ICIs post-ADC) may mitigate CRS while preserving efficacy [139, 140]. Biomarker-guided strategies (e.g. sST2/IL-6 monitoring) could further optimize safety [141–143]. Future protocols must balance efficacy with tailored toxicity management to expand the therapeutic window.

## IME framework: a multidimensional approach

**Figure 3:**
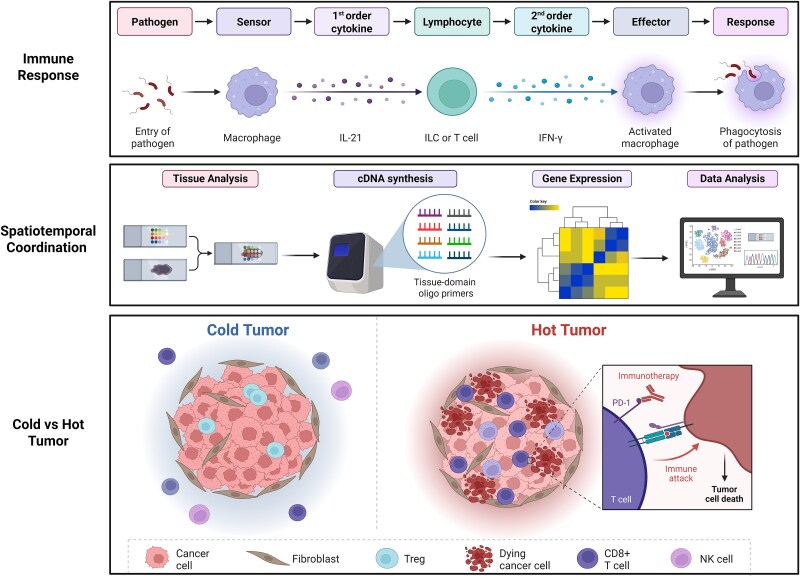
Spatiotemporal orchestration of tumor microenvironment reprogramming from innate activation to adaptive immune expansion and cold-to-hot conversion.

The concept of immune microenvironment reprogramming has evolved as a fundamental strategy in cancer immunotherapy, with early work demonstrating how targeted interventions can alter the immunosuppressive tumor milieu [144–146]. In colorectal cancer, metabolic reprogramming creates a nutrient-depleted, acidic microenvironment that suppresses immune function [144]. Similarly, breast cancer studies have established the paradigm of converting “cold” immune-excluded tumors into “hot” immune-infiltrated tumors through the modulation of immune cell populations and checkpoint molecules [145]. More recently, nanotechnology approaches have enabled precise IMER in challenging contexts like glioblastoma, where blood–brain barrier penetration and sequential targeting are required [146]. Building on these foundational concepts, our IMER framework represents a multidimensional strategy to optimize the synergistic interaction between ADCs and ICIs by systematically reprogramming the TME, which integrates innate immune activation, adaptive immune amplification, and spatiotemporal coordination, aiming to convert immunologically “cold” tumors into “hot” states while mitigating toxicity (Fig. 3).

IMER harnesses ADCs to initiate innate immune responses through ADCC and ICD. ADCC recruits NK cells and macrophages to tumor cells, releasing DAMPs (HMGB1, ATP, CRT) that activate DCs via TLR4/cGAS–STING pathways, enhancing antigen cross-presentation and CD8+ T-cell priming [7, 93]. Concurrently, ICD induces IFN-γ secretion, upregulating PD-L1 on tumor cells and myeloid cells—a feedback mechanism that ICIs (anti-PD-1/PD-L1) exploit to sustain T-cell function [147, 148]. This dual mechanism overcomes key immunosuppressive barriers, including Treg dominance and M2 macrophage polarization, while promoting T-cell infiltration and clonal expansion [145, 149].

IMER employs a tiered biomarker strategy to monitor TME dynamics: (i) serum biomarkers (HMGB1 for tumor lysis, CXCL9/CXCL10 for DC migration, and sST2 for toxicity risk) provide real-time response assessment [113, 150, 151]; (ii) spatial biomarkers (multiplex IF/spatial transcriptomics) reveal intratumoral heterogeneity, with interface PD-L1 expression predicting ICI efficacy and TCF1+ memory T cells indicating durable responses [152–154]; and (iii) liquid biopsies (ctDNA clearance, TCR clonality) enable early identification of nonresponders and irAE-prone patients [155–157].

By treating the TME as a dynamic, targetable entity, IMER redefines combination therapy. The framework harmonizes ADC cytotoxicity, ICI-mediated immune activation, and precision biomarker guidance to overcome historical resistance patterns and expand the therapeutic index. Key advancements include (i) AI-driven models integrating multiomics data to predict spatiotemporal responses; (ii) microbiome modulation (e.g. probiotics enhancing IFN-γ signaling); and (iii) modular ADCs codelivering checkpoint inhibitors for the suppression of localized immunosuppression.

## Future directions: multidimensional optimization strategies for ADC–ICI synergistic integration

The advancement of ADC–ICI synergistic integration hinges on three critical dimensions: (i) optimizing therapy chronology to maximize antigen release before checkpoint blockade, (2) engineering next-generation ADC payloads to enhance ICD, and (iii) implementing microenvironment normalization strategies to address spatial heterogeneity. Preclinical studies demonstrate that ADC priming prior to ICI administration significantly improves T-cell priming through enhanced DAMP release and DC maturation [158–160]. The phase III DREAMM 7 trial (NCT04246047) evaluates belantamab mafodotin plus bortezomib/dexamethasone (arm A) versus daratumumab with bortezomib/dexamethasone (arm B) in relapsed/refractory multiple myeloma, showing that lead-in ADC dosing reduces grade ≥ 3 irAEs by 40% while maintaining progression-free survival (PFS) benefits, though solid tumor validation remains pending [161–165].

Rational payload engineering represents another key advancement, with next-generation ADCs designed to amplify ICD. Pyrrolobenzodiazepine (PBD) dimers induce DNA cross-linking to enhance CRT exposure and STING pathway activation, while topoisomerase I inhibitors (e.g. deruxtecan payloads) increase cytosolic DNA accumulation to boost cGAS–STING signaling [166–168]. ADCT-601, a glycoengineered ADC targeting AXL receptor tyrosine kinase, utilizes Glycoconnect™ technology to conjugate a humanized IgG1 monoclonal antibody with a PBD dimer payload. Given AXL’s role in driving tumor progression and treatment resistance across multiple cancers (including lung, breast, pancreatic, esophageal, and hematologic malignancies) [169, 170], this ADC employs AXL-mediated cellular uptake, protease-mediated linker cleavage, and PBD-induced DNA interstrand cross-links to induce mitotic blockade and apoptosis. The phase I trial (NCT03700294) employs a 3 + 3 dose-escalation design to determine the maximum tolerated dose/recommended expansion dose, followed by tumor-specific expansion cohorts, prioritizing AXL-high populations to optimize therapeutic efficacy [171].

To address spatial heterogeneity, emerging TME remodeling strategies show promise in clinical settings. Anti-angiogenic therapies like bevacizumab (anti-VEGF-A) normalize tumor vasculature, reducing interstitial fluid pressure and improving ADC penetration into hypoxic tumor cores [172, 173]. The phase III IMpower150 trial (NCT02366143) evaluates atezolizumab (anti-PD-L1) combined with carboplatin/paclitaxel ± bevacizumab versus standard chemotherapy in Stage IV nonsquamous non-small cell lung cancer (NSCLC), demonstrating that PD-L1/vascular endothelial growth factor (VEGF) dual targeting improves PFS in high-tumor mutational burden subgroups (arm B: 9.7 vs arm C: 6.1 months, HR = 0.62) [174–176]. This supports the rationale for combining anti-PD-L1 and anti-VEGF therapies to overcome resistance in immunologically “cold” tumors, highlighting the potential of microenvironment modulation to enhance ADC–ICI combinations.

The development of bispecific ADCs represents an important evolution in targeted cancer therapy that aligns well with the principles of immune microenvironment reprogramming. These innovative agents combine the precision targeting capabilities of bispecific antibodies with the cytotoxic potency of traditional ADCs, offering several advantages for modulating the tumor immune landscape. By simultaneously engaging two distinct TAAs, bispecific ADCs demonstrate enhanced tumor selectivity while minimizing off-target effects on healthy tissues. This dual-targeting approach helps address the challenge of tumor heterogeneity where variable antigen expression often limits the efficacy of single-target therapies. The bispecific binding architecture also facilitates improved tumor penetration, overcoming diffusion barriers that frequently restrict conventional ADC distribution within solid tumors.

The therapeutic potential of bispecific ADCs integrates seamlessly with the multidimensional immune microenvironment reprogramming framework. Their ability to simultaneously target tumor antigens while engaging immune cell receptors creates localized immune activation hubs that complement the immunogenic cell death induced by cytotoxic payloads. This coordinated action bridges innate and adaptive immunity more effectively than sequential administration of separate therapies. The simultaneous targeting capabilities offer inherent advantages for addressing spatial compartmentalization challenges in TMEs, potentially overcoming physical barriers that limit immune cell infiltration.

While bispecific ADCs show considerable promise, several important considerations emerge for their clinical translation. The increased molecular complexity necessitates careful evaluation of safety profiles, particularly regarding potential cytokine release syndromes and other irAEs. Developing predictive biomarkers will be crucial for appropriate patient selection given the dual-targeting nature and complex TME interactions. Future research directions should explore optimal combination strategies with existing immunotherapies and investigate potential resistance mechanisms to maximize therapeutic durability. As this field advances, continued investigation into clinical applications and integration with current treatment paradigms will be essential for realizing the full potential of bispecific ADCs in cancer immunotherapy.

## Conclusion

The combination of ADCs and ICIs represents a paradigm shift in oncology, overcoming single-agent limitations through synergistic TME reprogramming. ADCs trigger innate immunity via ADCC and ICD, releasing TAAs and DAMPs that recruit cytotoxic effectors and enhance antigen presentation. This innate activation primes adaptive responses through IFN-γ-mediated PD-L1 upregulation and TCR clonal expansion, which ICIs then sustain by blocking checkpoint pathways. However, spatial heterogeneity (perivascular ADC accumulation vs hypoxic “deserts”) and temporal dynamics critically influence this synergy, as evidenced by enhanced responses in refractory cancers (DESTINY-PanTumor02 and KEYNOTE-355) despite ongoing challenges in biomarker optimization and toxicity management.

The IMER framework addresses these challenges through a multidimensional approach integrating longitudinal biomarkers (HMGB1, CXCL9 gradients) and spatial mapping (PD-L1 interface patterns, TCF1+ memory T cells) to guide ADC–ICI sequencing. Preclinical advances in glycoengineered ADCs (e.g. ADCT-601) and vascular normalization strategies (e.g. bevacizumab combinations) demonstrate therapeutic potential, though clinical translation requires addressing overlapping toxicities (CRS, pneumonitis) and refining patient stratification via composite biomarkers. Future directions should focus on three key pillars: (i) chronological coordination of therapy to maximize antigen release before checkpoint blockade, (ii) next-generation payload engineering to enhance ICD, and (iii) microenvironment modulation to overcome spatial barriers. By harmonizing ADC cytotoxicity, ICI-mediated immune activation, and precision biomarker guidance, IMER offers a transformative approach to converting immunologically “cold” tumors into “hot” states, expanding the therapeutic index while mitigating toxicity—ultimately redefining combination immunotherapy design and overcoming historical resistance patterns in oncology.

## Data Availability

All the raw data supporting the findings of this study are available from the corresponding authors upon reasonable request. The publicly available datasets referenced in the manuscript (e.g. TCGA and GEO) are cited in the reference list.
